# Supplementary material for: Experimental evolution of yeast shows that public-goods upregulation can evolve despite challenges from exploitative non-producers
Source: Nat Commun. 2024 Sep 6;15:7810. doi: 10.1038/s41467-024-52043-9 (PMC11379824; doi:10.1038/s41467-024-52043-9)

Supplementary Information

Experimental evolution of yeast shows that public-goods upregulation can evolve despite challenges from exploitative non-producers

Richard J Lindsay[1], Philippa J Holder[1], Mark Hewlett[1] and Ivana Gudelj[1]*

[1]Biosciences and Living Systems Institute, University of Exeter, Exeter, EX4 4QD, UK

*Corresponding author: I.Gudelj@exeter.ac.uk

RJL: R.J.Lindsay@exeter.ac.uk,

PJH: P.J.Holder@exeter.ac.uk,

MH: M.Hewlett@exeter.ac.uk.

Contents:

Supplementary Figures 1-15

Source Data: Uncropped gel and blot scans

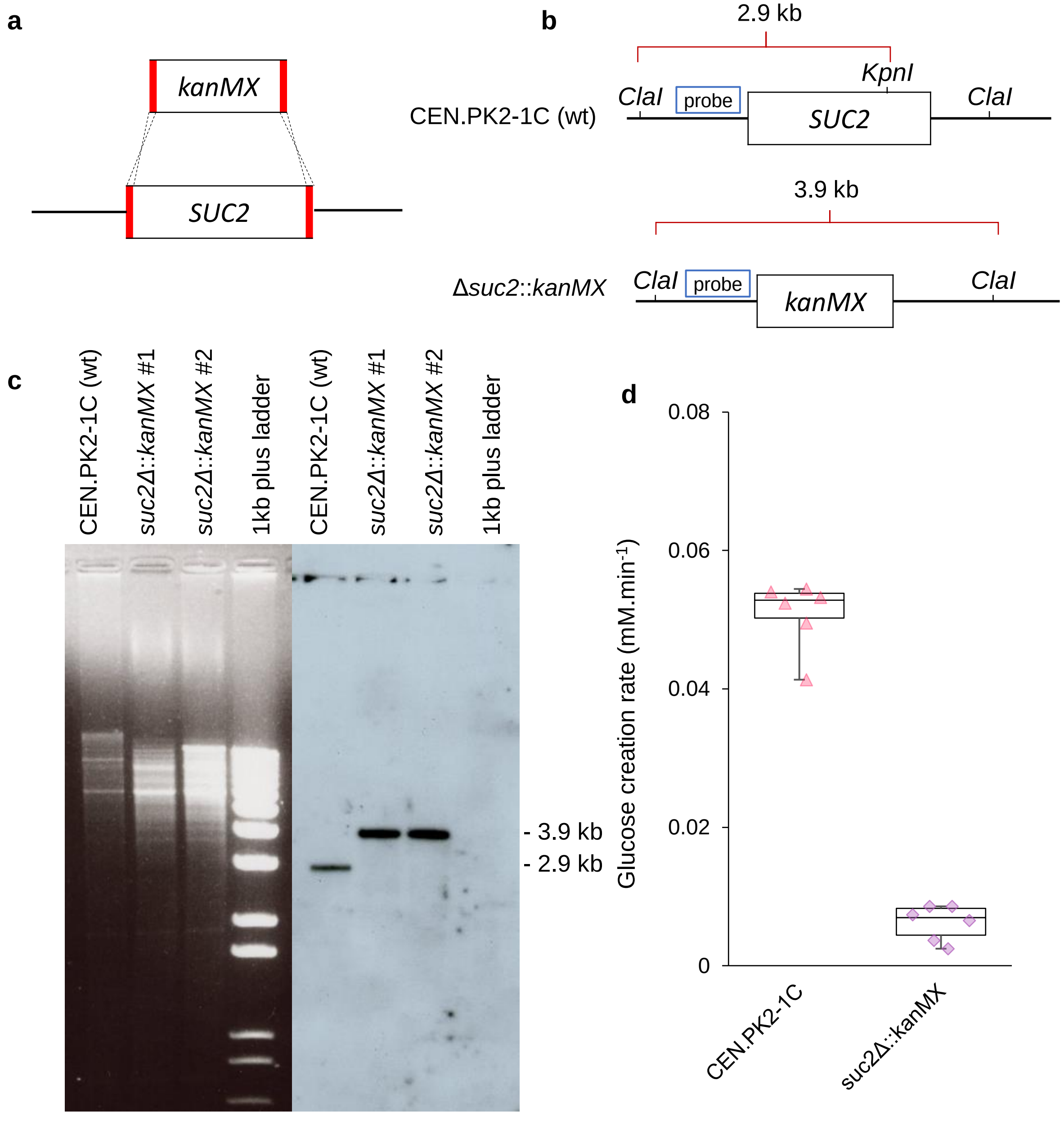

Supplementary Figure 1: Generating a more competitive non-producer. a a *SUC2*-deletion strain was developed by generating a deletion vector by PCR amplification of the geneticin (G418) resistance marker (*kanMX*) containing extensions with homology to 5' and 3' UTR beyond the *SUC2* ORF (indicated in red). **b** Transformants conferring geneticin resistance were tested for gene replacement with a 700 bp digoxigenin-labelled probe (Roche) from a region upstream of the *SUC2* ORF that was visualised with CDP-*Star* Chemiluminescent Substrate (Sigma). **c** gDNA was restriction digested with *ClaI and KpnI,* resulting in a 2.9 kb band for wt and a 3.9 kb band for gene replacement mutants. Transformants #1 and #2 were both positive for gene replacement (Δ*suc2*::*kanMX*). **d** Enzymatic assays confirmed that gene replacement resulted in a significant reduction in invertase activity (Two-sample, two-sided t-test: $t = 19.54$, $p < 2.70 \times 10^{-9}$). Box-plots show 25, 50, 75th percentiles, whiskers show min/max, points show all replicates, $n = 6$. All data are provided in the Source Data File.

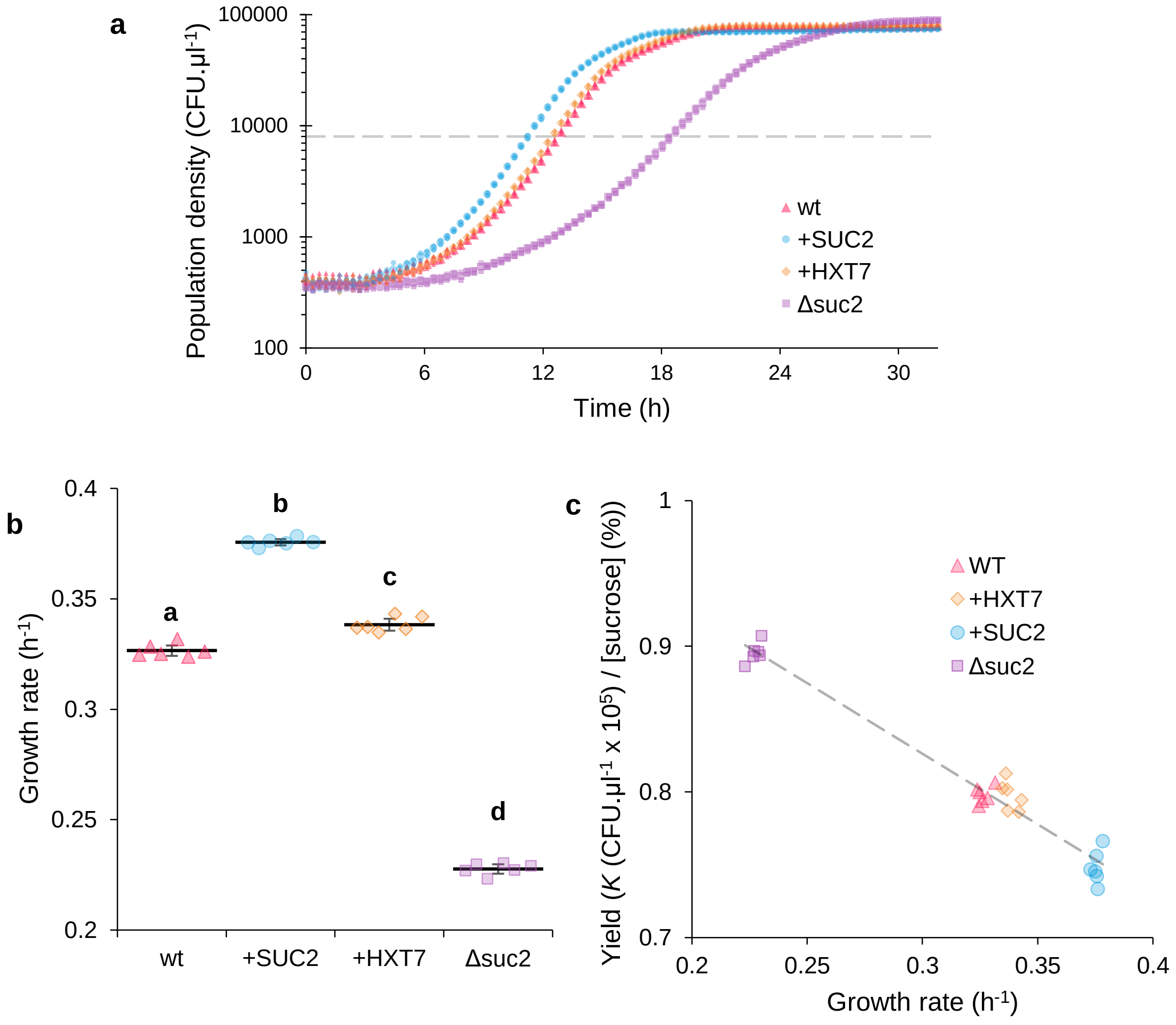

Supplementary Figure 2: Growth properties of strains with different sucrose metabolic properties on 1% sucrose SC media. wt is CEN.PK2-1C ("producer"), +SUC2 and +HXT7 overexpress invertase (SUC2) or a hexose transporter (HXT7), Δsuc2 is the invertase deletion mutant (non-producer). Strains were axenically grown at an initial density of approximately 10^2.µl^{-1} (with precise densities established by flow cytometry). Growth rates were calculated as Malthusian growth parameters (*w*) up to the time-point where density exceeded 8 x 10^3.µl^{-1} (>6 doublings, indicated by dashed horizontal lines). **a** Time series of population density measured by absorbance (620 nm). Points show mean ± 95% C.I., n = 6. N.B. Population densities below approximately 5 x 10^2.µl^{-1} are not accurately captured by this measure so initial densities were quantified by flow cytometry. **b** Growth rate was significantly associated with genotype (Linear model: $F_{(3,20)}$ = 3190, $p < 2.2 \times 10^{-16}$, Adj. R^2 = 0.998). Letters indicate significant differences between genotypes (Tukey multiple comparison of means (HSD method): $p < 1.98 \times 10^{-6}$) Lines show mean ± 95% C.I.. **c** A between-strain rate-yield trade-off was found (Linear model (shown by dashed line): β = -9.69 x 10^4, $F_{(1,22)}$= 615.9, $p < 2.2 \times 10^{-16}$, Adj, R^2 = 0.964). Carrying capacity (*K*) was the population density at 32 h. **b**-**c** points show all replicates, n = 6 (per genotype). All data are provided in the Source Data File.

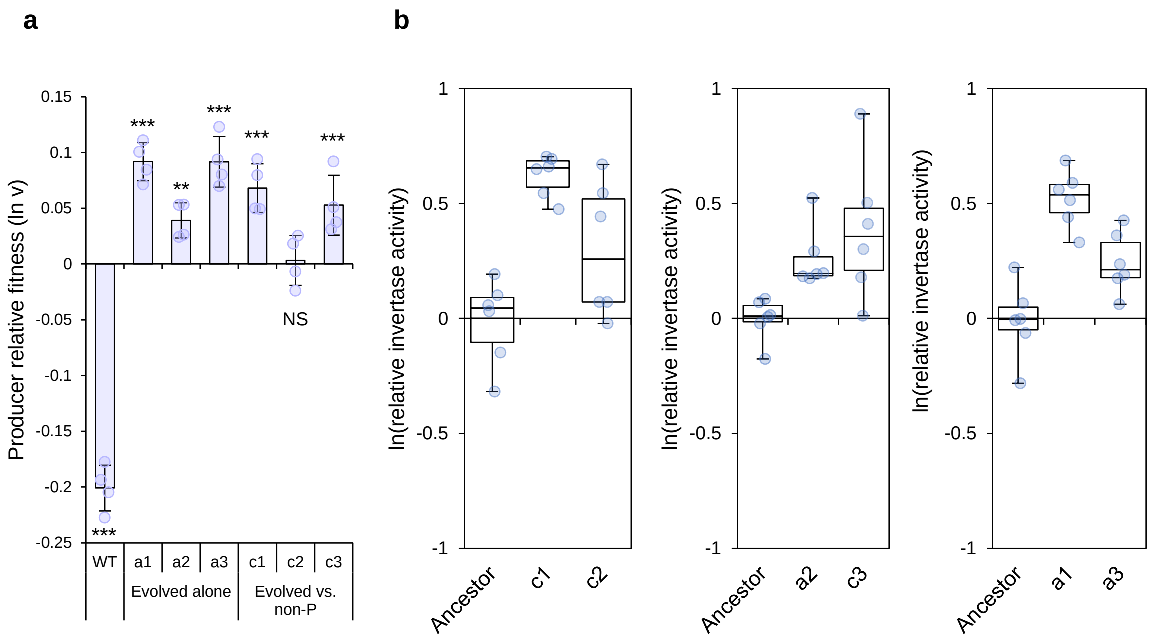

Supplementary Figure 3: The relative fitness and invertase activity of evolved producers. a Relative fitness (ln *v*) of WT (ancestor) and producer strains evolved in the presence or absence of non-producers when in pairwise competition with ancestral non-producers over a 24h season (equal fitness = 0). Significantly different fitness from non-producers is shown (*** $p < 0.001$, ** $p < 0.01$, NS $p > 0.05$, linear model: $p < 3.57 \times 10^{-13}$, $F_{(2,25)} = 111.3$, Adj. $R^2 = 0.891$). Bars show mean ± 95% C.I., n = 4. **b** Comparisons of invertase activity of evolved strains compared to the ancestral producer. For practicality and to standardise measures between different experiments, three strains were assessed for invertase activity simultaneously and invertase activity was normalised to the mean ancestral producer activity that was being concurrently measured. Relative invertase acitivity was ln-transformed for statistical analysis. All evolved strains had elevated invertase activity compared to the ancestor (Linear model: $p < 0.05$; see Supplementary Data 1 for data analysis). Box-plots show 25, 50, 75th percentiles, whiskers show min/max, points show all replicates, n = 6. All data are provided in the Source Data File.

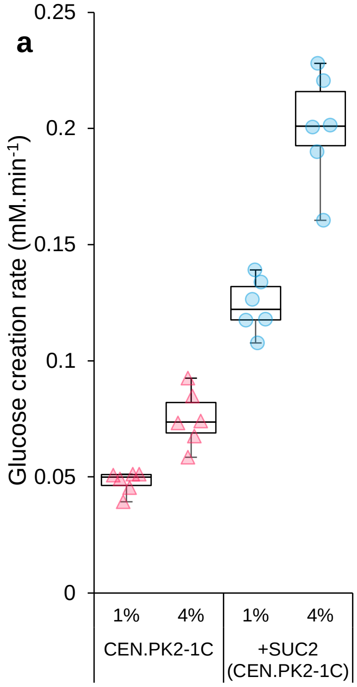

b

GLM: Glucose creation rate was significantly associated with genotype and sucrose concentration (as factor) ($F_{(3,20)} = 121.7$, $p < 5.15 \times 10^{-13}$, Adj. $R^2 = 0.940$).

Specifically:

- +SUC2 had a higher rate than CEN.PK2-1C ($p < 2.30 \times 10^{-8}$)
- Rate increased with sucrose concentration ($p < 4.64 \times 10^{-3}$) and this association was stronger for +SUC2 than for CEN.PK2-1C ($p < 6.18 \times 10^{-4}$).

Tukey multiple comparison of means (HSD method):

Strain 1 ([sucrose])	< Strain 2 ([sucrose])	p-value
CEN.PK2-1C (1%)	< CEN.PK2-1C (4%)	0.0221
+SUC2 (1%)	< +SUC2 (4%)	1.18×10^{-7}
CEN.PK2-1C (1%)	< +SUC2 (1%)	1.31×10^{-7}
CEN.PK2-1C (4%)	< +SUC2 (4%)	2.31×10^{-11}

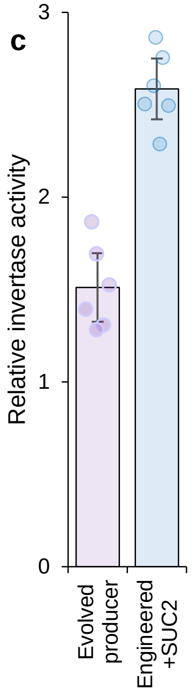

Supplementary Figure 4: Invertase activity of the engineered SUC2 overproducing strain +SUC2. **a** Invertase activity of +SUC2 was compared to its otherwise isogenic progenitor (CEN.PK2-1C). Expression was induced in 0.01 % glucose SC media and then measured as hexose creation (glucose is treated as equivalent to fructose) in either 1% or 4% sucrose (see methods for more details). Box-plots show 25, 50, 75th percentiles, whiskers show min/max, points show all replicates, n = 6. **b** Summary of statistical analysis. **c** +SUC2 had relatively higher SUC2 activity than the evolved producers (Two-sample, two-sided t-test: $t = 8.50$, $p < 6.91 \times 10^{-6}$, $n = 6$). Each evolved producer value is the mean of 6 technical replicates for each of the 6 tested evolved strains in Supplementary Fig. 3b. Engineered +SUC2 values in **c** are from **a** (1% sucrose). All data are provided in the Source Data File.

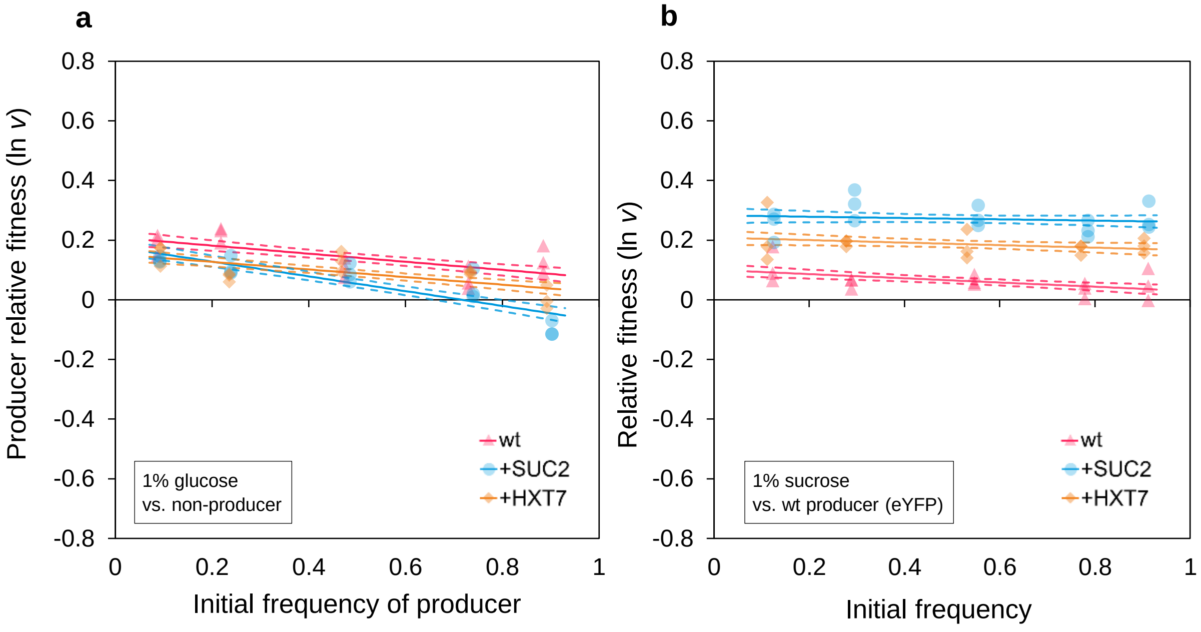

Supplementary Figure 5: Relative fitness of producer strains. a Fitness differences on 1% sucrose media (Fig. 3a) were determined using a GLM with a genotype:frequency interaction term ($F_{(5,39)} = 69.24$, $p < 2.2 \times 10^{-16}$, Adj. $R^2 = 0.886$; specific between-genotype differences are shown in Fig. 3). These differences were confirmed to be caused by properties of sucrose metabolism by conducting equivalent competitions (non-producer vs. wt, +HXT7 or +SUC2), but in 1% glucose media. Outcomes were analysed using the same modelling approach as in 1% sucrose (GLM: $F_{(5,39)} = 16.24$, $p < 1.21 \times 10^{-8}$, Adj. $R^2 = 0.634$). On 1% glucose, the fitness differences were not significant (wt c.f. +HXT7: $p = 0.100$, wt c.f. +SUC2: $p = 0.353$, +HXT7 c.f. +SUC2: $p = 0.464$). **b** wt producer, +HXT7 or +SUC2 (mCherry) were competed pairwise against the wt producer (*eYFP*) in 1% sucrose. +SUC2 ($p < 3.39 \times 10^{-6}$) and +HXT7 ($p < 2.35 \times 10^{-3}$) were stronger competitors than the wt producer (GLM: $F_{(5,39)} = 34.0$, $p < 3.22 \times 10^{-13}$, Adj. $R^2 = 0.790$). Points show all replicates, n = 3 for each frequency/competition combination. Lines show a linear model (± S.E.) for individual genotypes. All data are provided in the Source Data File.

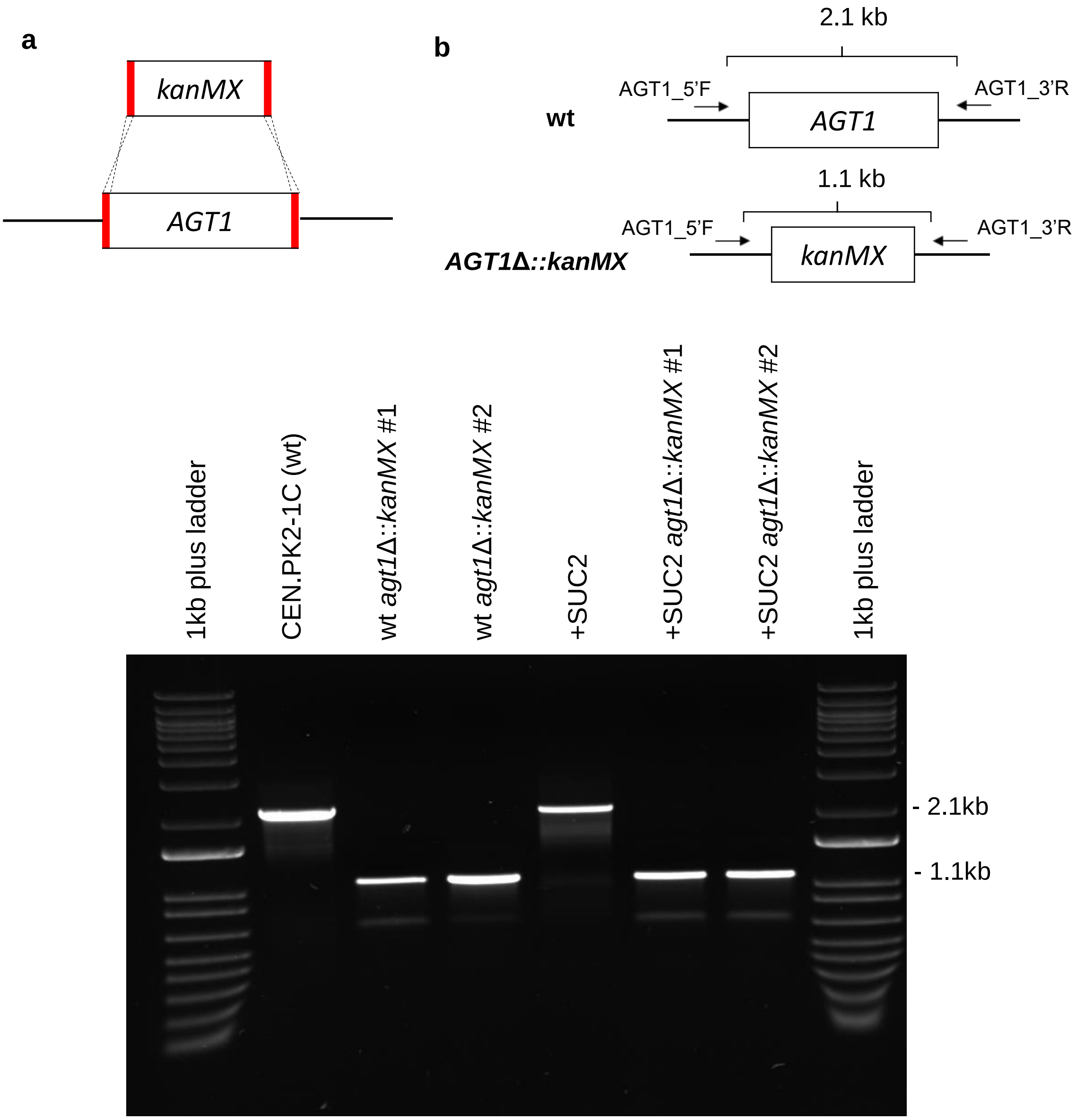

Supplementary Figure 6: Generating producers with sucrose transporter (*AGT1*) deletions. a *AGT1* deletion strains of wt (CEN.PK2-1C) or the invertase overexpressing strain (+SUC2) were developed by generating a deletion vector by PCR amplification of the geneticin (G418) resistance marker (*kanMX*) containing extensions with homology to 5' and 3' UTR beyond the *AGT1* ORF (indicated in red). **b** Transformants conferring geneticin resistance were tested for gene replacement by PCR of gDNA using primers (AGT1_5'F & AGT1_3'R) that bound outside of the vector sequence to produce a 2.1 kb amplicon for the wt allele and 1.1 kb for positive gene replacement (*agt1*Δ::*kanMX*)

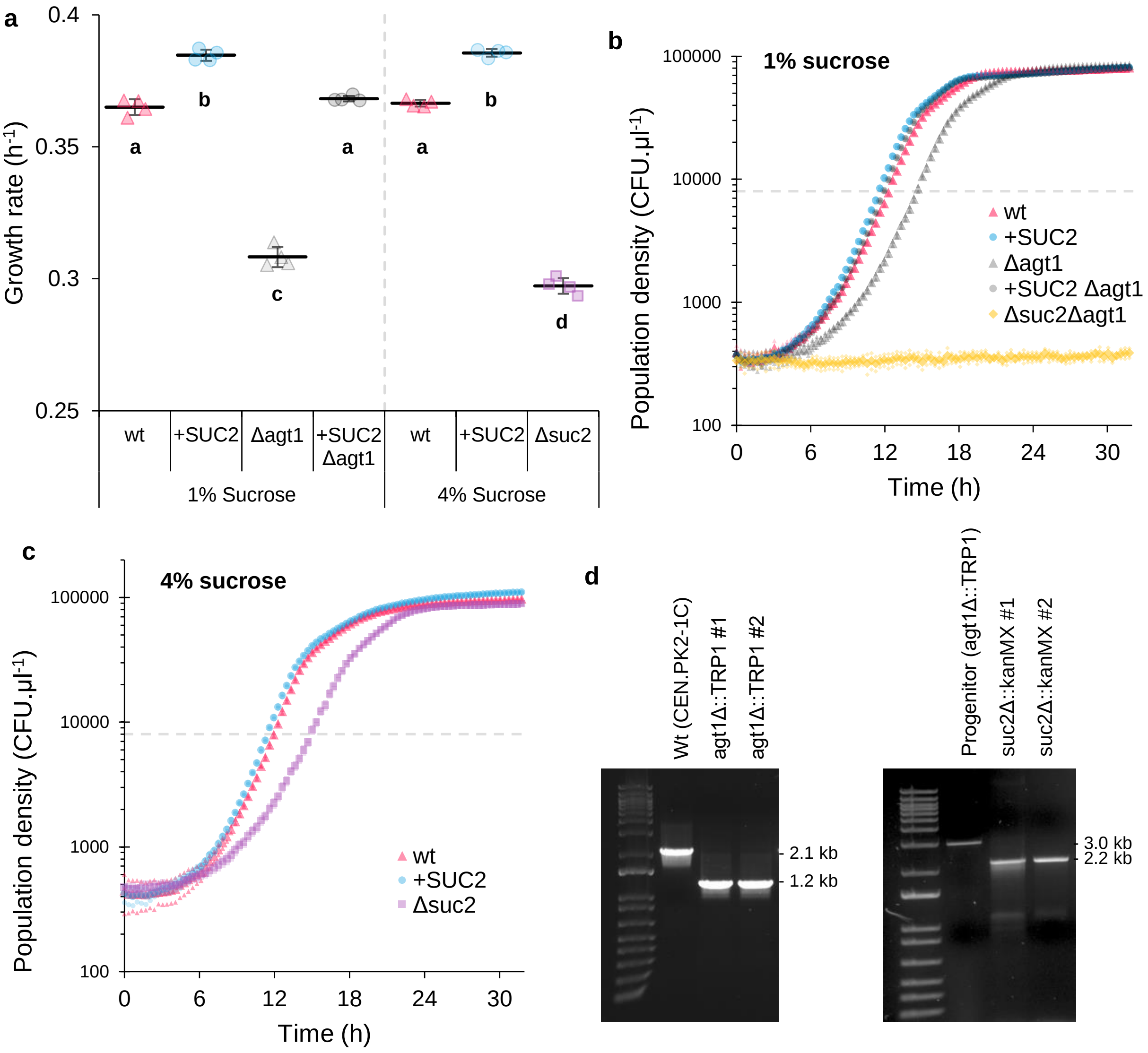

Supplementary Figure 7. Growth rates of strains with different sucrose metabolic properties on SC media with different sucrose concentrations. Strains were grown axenically at an initial density of approximately $10^2.\mu l^{-1}$. **a** Growth rates were calculated as Malthusian growth parameters (*w*) (mean ± 95% C.I., n = 4) up to the time-point where density exceeded 8 x $10^3.\mu l^{-1}$ (at least 6 doublings, indicated by dashed horizontal lines in **b**-**c**). Different letters indicate significant differences in *w* (GLM: $F_{(6,21)}$= 838.2, p < 2.2 x 10^{-16}, Adj. R^2= 0.995, with post-hoc Tukey's (HSD) multiple comparison of means, p < 0.001, NS: p > 0.05). **b**-**c** Show time series of population density measured by absorbance (620 nm). Large points show mean ± 95% C.I. (small points – often obscured by mean), n = 4. N.B. Population densities below approximately 5 x $10^2.\mu l^{-1}$ are not accurately captured by this measure so initial densities were quantified by flow cytometry. **b** = 1%, **c** = 4% sucrose. **d** The *AGT1/SUC2* double mutant was confirmed by PCR. *AGT1* was replaced by *TRP1* and verified as described in Supplementary Fig. 6b (left) but successful replacement (agt1Δ::TRP1) producing a 1.2 kb amplicon (wt = 2.1 kb). *SUC2* replacement was as described in Supplementary Fig. 1 but was verified by PCR using primers SUC2_5'F and SUC2_3'R giving an amplicon of 2.2 kb for successful replacement (suc2Δ::kanMX, right, wt = 3.0 kb). All data are provided in the Source Data File.

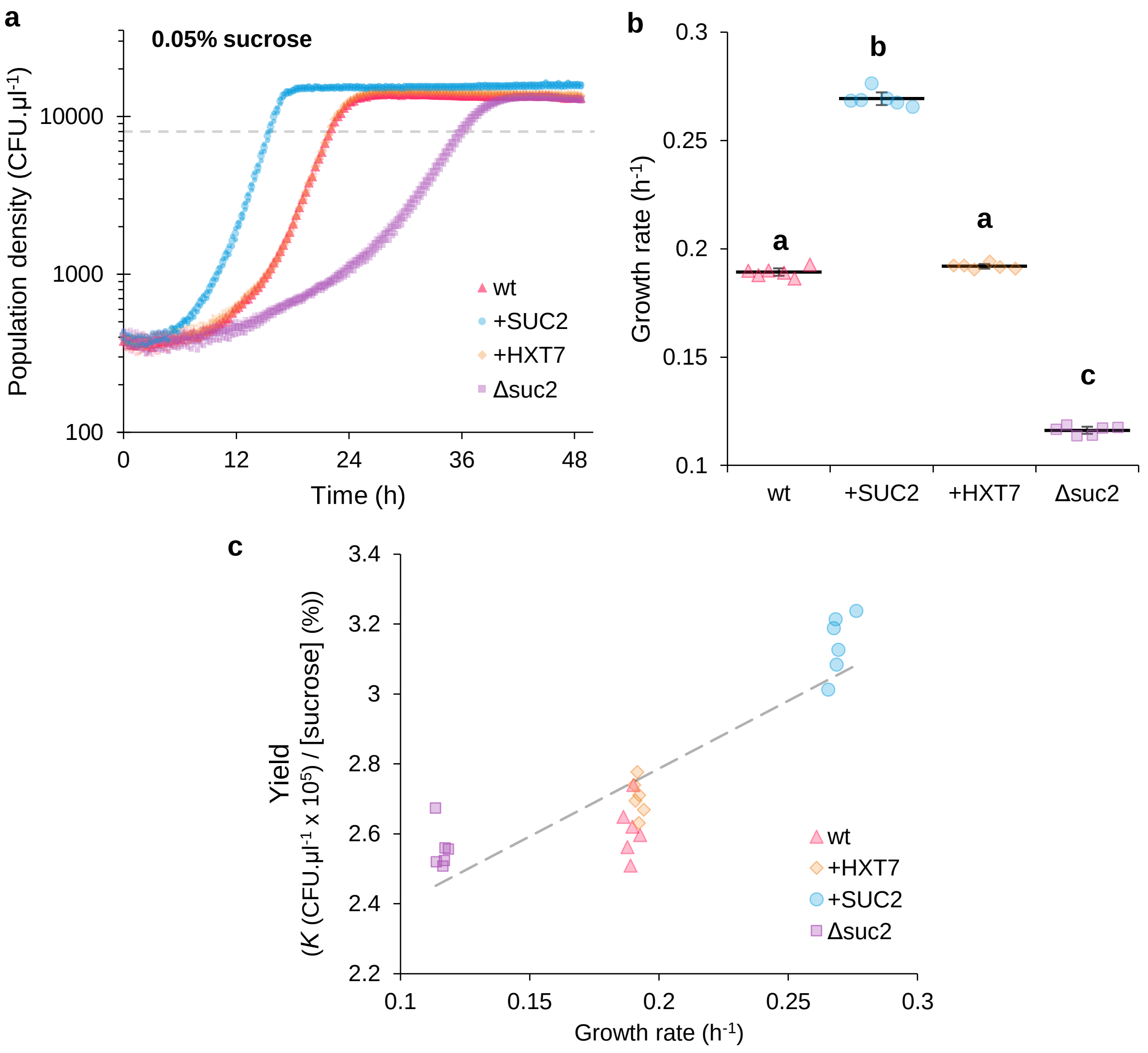

Supplementary Figure 8. a-c Strains were grown axenically in 0.05% sucrose media, initial density = approximately 10^2.μl^{-1}. **a** Population density time series measured by absorbance (620 nm). Large points show mean ± 95% C.I. (small points – often obscured by mean), n = 6. N.B. Population densities below approximately 5 x 10^2.μl^{-1} are not accurately captured by this measure so initial densities were quantified by flow cytometry for calculating *w*. **b** Growth rates were calculated as Malthusian growth parameters (*w*) (mean ± 95% C.I., n = 6) up to the time-point where density exceeded 8 x 10^3.μl^{-1} (at least 6 doublings, indicated by dashed horizontal line in **b**). Different letters indicate significant differences in *w* (GLM: $F_{(3,20)}$= 3892, $p < 2.2 \times 10^{-16}$, Adj. R^2= 0.998, with post-hoc Tukey's (HSD) multiple comparison of means, $p < 0.001$, NS: $p > 0.05$). **c** Despite growth rate differences in these conditions, the between-strain rate-yield trade-off observed in 1% sucrose (Supplementary Fig. 2c) was not detected in 0.05% sucrose. Instead, a positive relationship between growth rate and yield was detected (Linear model (shown by dashed line): $\beta = 3.88 \times 10^5$, $F_{(1,22)}$= 73.3, $p < 1.89 \times 10^{-8}$, Adj, R^2 = 0.759), thought to be caused by maintenance energy requirements. Carrying capacity (*K*) is final population density in **a**. All data are provided in the Source Data File.

Supplementary Figure 9

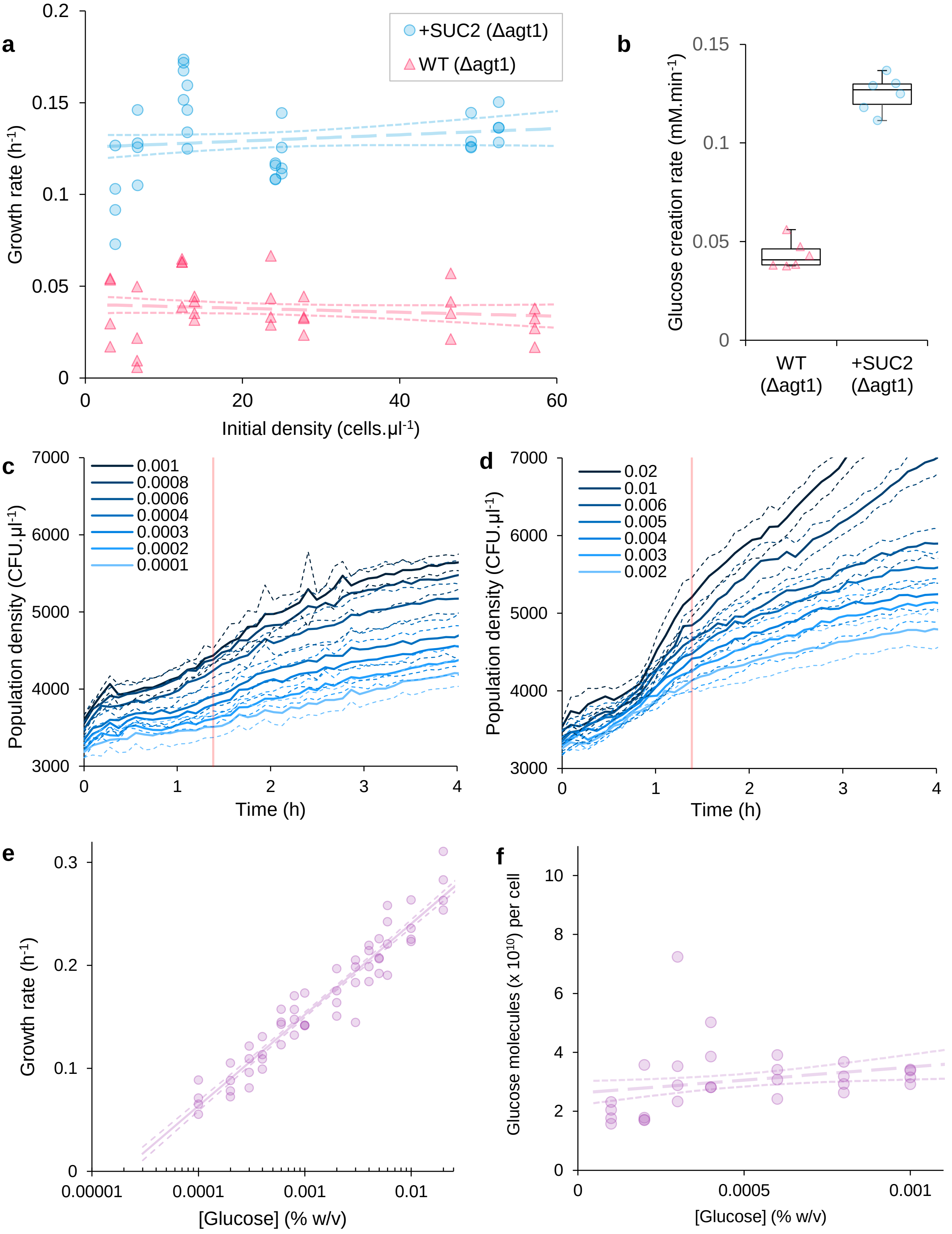

Supplementary Figure 9. Estimating glucose capture efficiency.

a Growth rate (*w*) of populations where initial densities were below 60.μl^{-1}, where density-dependent growth rates were not detected (GLM (linear model ± s.e.): Initial density: WT: p = 0.502; +SUC2 = 0.471), markers show all replicates. **b** Invertase activity of strains directly from overnight starter culture conditions. Box-plot shows 25, 50, 75th percentiles, whiskers show min/max, points show all replicates, n = 6. Expression levels were equivalent to the de-repressed conditions (Supplementary Fig. 4). **c-e** Growth rates (*w*) were measured over approx. 83 minutes after inoculation (indicated by vertical red line) in differing glucose concentrations (0.0001 % - 0.02 % w/v)**. c-d** Show population density time series mean (solid line) ± 95% C.I. (dashed lines), n = 4. **e** Growth rate increases with glucose concentration (log-model ± s.e.), n = 4 for each concentration. Markers show all replicates. **f** The number of glucose molecules required per cell grown was estimated from the maximum densities (over 24h) of populations in **c**. A rate-efficiency trade-off was not detected over these concentrations (linear model ± s.e.: p = 0.222). n = 4 for each concentration. Markers show all replicates. All data are provided in the Source Data File.

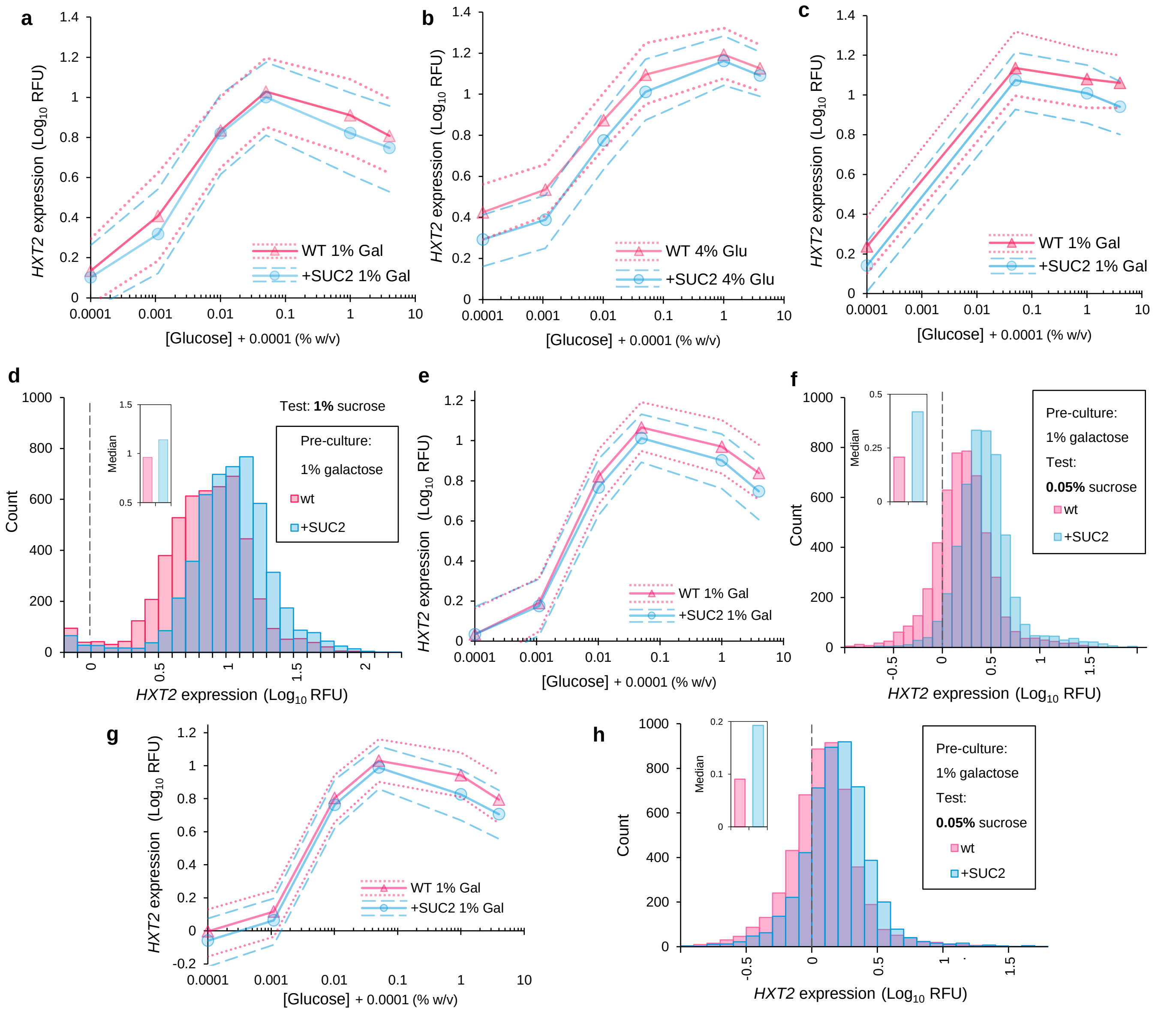

Supplementary Figure 10. *HXT2* expression was compared between the WT and +SUC2 using *HXT2*-promoter-regulated *EGFP*. Cells were pre-cultured in repressing conditions: either SC media lacking glucose (1% galactose – **a**,**c**,**e**,**g**) or in high glucose (4% - **b**) before being transferred to SC media containing the indicated glucose concentrations and incubated for 3 h to induce expression. As previously (Ozcan & Johnson, 1995), *HXT2* expression is initially induced by increasing glucose concentrations, but downregulated by higher glucose concentrations. This expression pattern was the same in both the WT and +SUC2, and was found when cells were pre-cultured in both 1% galactose (**a**,**c**,**e**,**g**) or 4% glucose (**b**). However, when tested in 1% sucrose, +SUC2 had significantly increased *HXT2* expression compared to the wt (Fig. 6b), and this increase was consistent both when starter cultures were supplemented with either repressing condition, 1% galactose or 4% glucose. **a**-**b** Show glucose-concentration-regulated expression profiles that accompany data shown in Fig. 6b. **c** Shows equivalent data that accompanies the experimental repeat shown in **d**, where equivalent expression pattens between strains were found to those in Fig. 6b. This difference was also found in 0.05% sucrose (**f** & **h**) following preculture in 1% galactose. **e&g** show glucose-concentration-regulated expression profiles that accompany **f**&**h**, respectively. **e**&**f** are experimental repeats of **g**&**h.** **a**-**c**,**e**,**g** Solid lines follow the median, dashed/dotted lines show 25/75th percentiles, n = 4388-5000 flow cytometry events (cells). **d**,**f**,**h** show expression distribution of 5000 cells per strain (Insets show median value of 5000 events). Vertical dashed line (=0) shows median signal from non-fluorescing wt, against which expression data was normalised. All data are provided in the Source Data File.

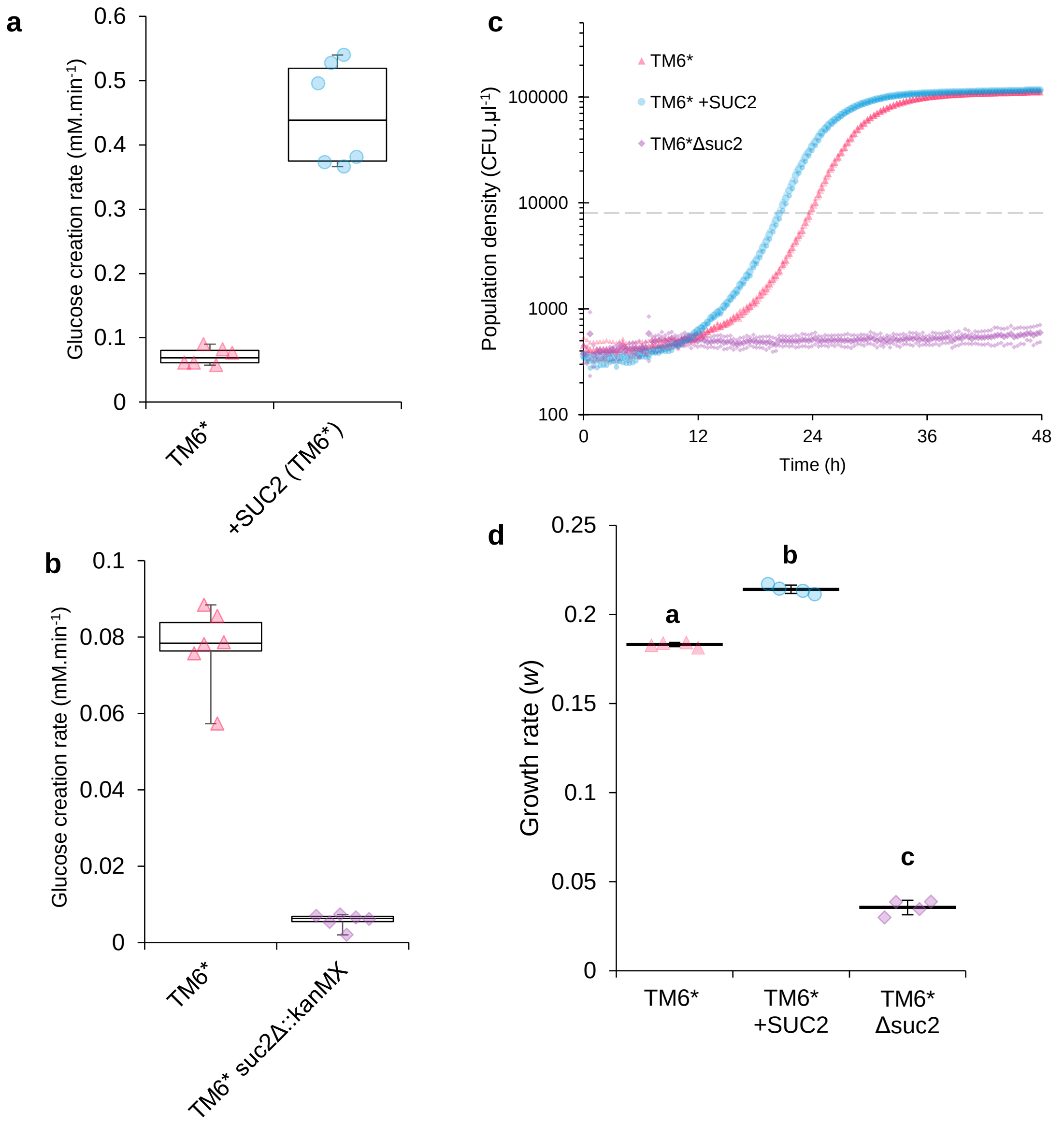

Supplementary Figure 11. a An extra copy of *SUC2,* regulated by the *GPD* promoter, was introduced into the TM6* genetic background. The resulting strain (TM6*+SUC2) had increased invertase activity (Welch's two-sided t-test: t = 11.1, $p < 1.06 \times 10^{-4}$). **b** A previously generated (Lindsay et al., 2018) suc2-deletion strain in the TM6* genetic background was confirmed to have significantly impaired invertase activity (Welch's two-sided t-test: t = 15.8, $p < 8.55 \times 10^{-4}$). **a**-**b** Box-plots show 25/50/75th percentiles, whiskers show min/max. Points show all replicates, n = 6. **c** Time series of population density measured by absorbance (620 nm). Large points show mean ± 95% C.I. (small points – often obscured by mean), n = 4. N.B. Population densities below approximately 5×10^2.µl^{-1} are not accurately captured by this measure so initial densities were quantified by flow cytometry for calculating *w*. **d** Growth rates were calculated as Malthusian growth parameters (*w*) (mean ± 95% C.I., points show all replicates, n = 4) up to the time-point where density exceeded 8×10^3.µl^{-1} (at least 6 doublings, indicated by dashed horizontal line in **b**), or after 48 h in the case of TM6*Δsuc2*.* *w* was significantly associated with genotype (GLM: $F_{(2,9)} = 4356$, $p < 3.65 \times 10^{-14}$, Adj. R^2= 0.999). Different letters indicate significant differences in *w* (TM6* < TM6*+SUC2: $p < 1.05 \times 10^{-7}$; TM6*Δsuc2 < TM6*: $p < 9.51 \times 10^{-14}$; TM6*Δsuc2 < TM6*+SUC2: $p < 1.72 \times 10^{-14}$). All data are provided in the Source Data File.

The influence of increasing hexose creation rates on +SUC2 axenic growth rate was tested by increasing resource concentration to 4%. Under these conditions (4%), sucrose hydrolysis rates were higher than in 1% sucrose, with +SUC2 having a greater difference in activity between 1% vs. 4% compared to the wt (Supplementary Fig. 4). Despite this increase, growth rates were not significantly different (Supplementary Fig. 7), which suggests that while hexose availability in the environment increases, either hexose uptake by the producer does not, or its benefits are offset by metabolic inefficiencies (Postma et al. 1989; Otterstedt et al. 2004) (Fig. 6b).

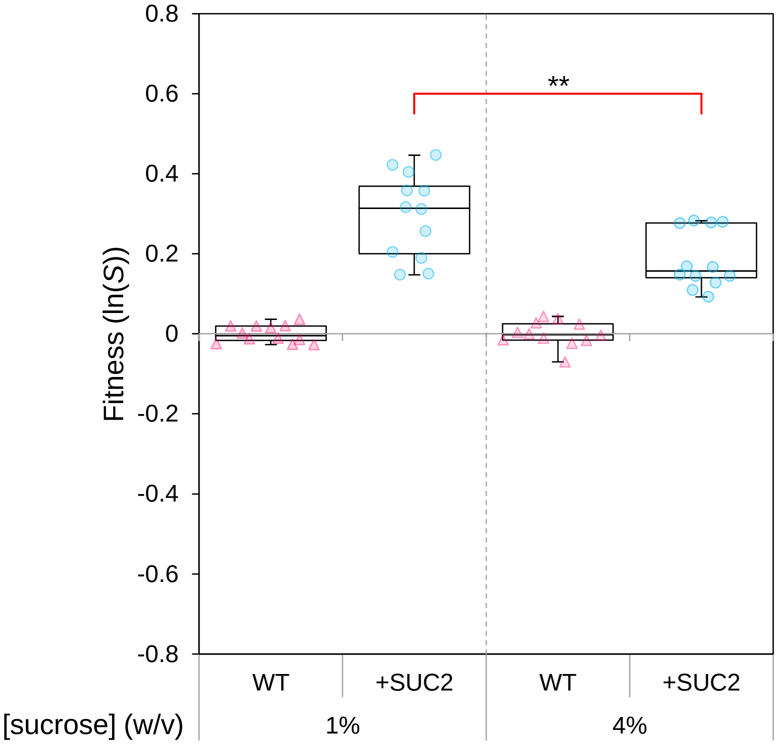

Supplementary Figure 12. As shown above, during competition experiments with non-producers, we found that +SUC2 was still fitter than wt (Tukey HSD: $p = 6.83 \times 10^{-6}$) but the selective advantage of +SUC2 was lower in 4% compared to 1% sucrose (Tukey HSD: $p = 7.13 \times 10^{-3}$) (1% data is the same as that shown in Fig. 5). We attribute this difference to non-producers capturing a larger proportion of hexose in 4% versus 1%, which are more readily available when competing against +SUC2 than the wt. The fitness difference of +SUC2 between 1% and 4% sucrose demonstrates how the competitiveness of overproducers is sensitive to the proportion of the generated hexose that they capture. Although, this provides some evidence to support Mechanism 2, this extra hexose capture by the non-producer might also impose reduced metabolic efficiency, as described in Mechanism 1. However, the lower competitiveness of +SUC2 in 4% versus 1% sucrose suggests the extra hexose capture benefits non-producers to a greater extent than any cost of lower efficiency. Points show all replicates, box-plots show 25, 50, 75th percentiles, whiskers show min/max, n = 12 collated from 3 experimental repeats. NS, not significant ($p > 0.05$), *** $p < 0.001$. full analysis shown in Supplementary Data 2. All data are provided in the Source Data File.

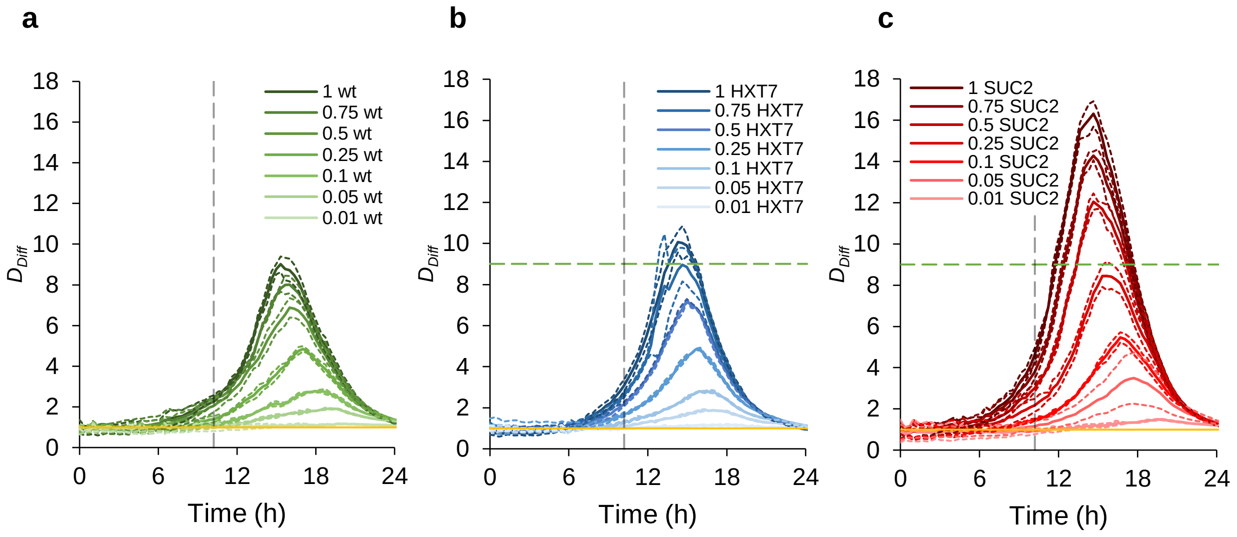

Supplementary Figure 13. To test how different producer genotypes support overall population growth when challenged by non-producers, the growth of populations (all initial density of $10^2.\mu l^{-1}$) initiated with different frequencies of non-producer and each producer genotype (**a** wt, **b** +HXT7, **c** +SUC2) were assessed in 1% sucrose SC media over 24h. Population density was assessed as absorbance (620 nm) and plotted relative to pure non-producer populations ($D_{Diff} = {}^{D_{pro}}/_{D_{non}}$, where D_{pro} is the density of producer-containing populations and D_{non} is the density of pure non-producer populations (pure non-producer = 1 (indicated by solid yellow horizontal line)) at each time-point. The maximum difference in relative density between the pure non-producer populations and each producer-containing population (D_{Diff}^{max}) is plotted in Fig. 7c. Time-points pre-10h (indicated with vertical dashed line) were excluded to omit noisy readings in low-density populations. Mean (solid lines) ± 95% C.I. (dashed lines), n = 3. Horizontal dashed lines in **b**-**c** indicate the mean maximum relative difference of pure wt populations. All data are provided in the Source Data File.

Supplementary Figure 14

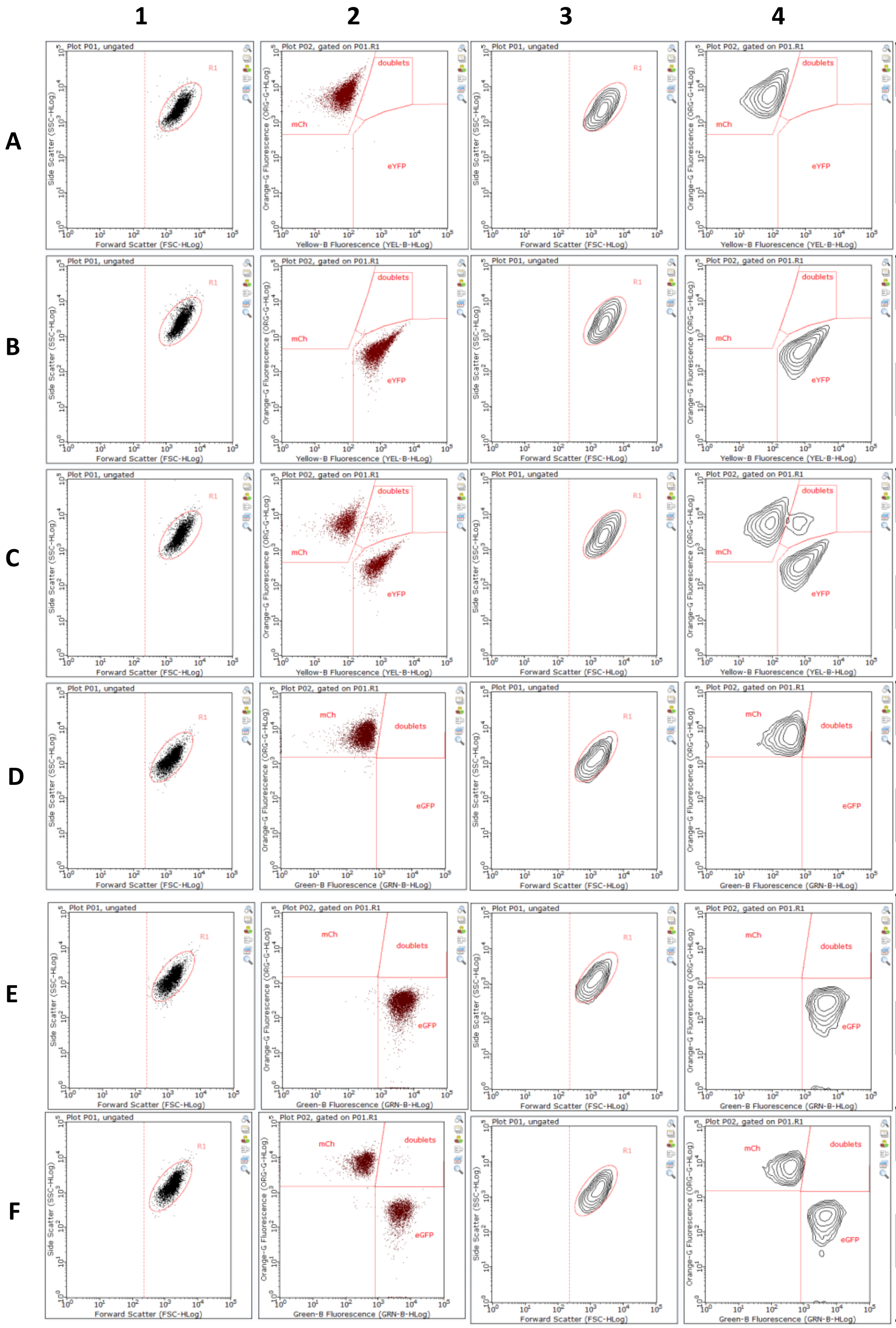

5

Region	%
R1	99.24
mCh	99.86
eYFP	0.02
doublets	0.04
R1	99.30
mCh	0.00
eYFP	99.82
doublets	0.10
R1	99.24
mCh	44.14
eYFP	52.53
doublets	3.35
R1	99.16
mCh	99.66
eGFP	0.00
doublets	0.00
R1	98.82
mCh	0.02
eGFP	99.62
doublets	0.00
R1	99.24
mCh	50.10
eGFP	48.95
doublets	0.71

Supplementary Figure 14. Genotype frequencies were measured during competition experiments by flow cytometry of strains that expressed different fluorescent protein markers using a Guava easyCyte 10HT System using Guava InCyte software version 3.2 (Merck Millipore). Examples of the gating strategy used are shown. Populations were gated (R1) on Forward scatter (FSC) against Side scatter (SSC) measurements (Columns **1** & **3**). Events in R1 were then differentiated based on fluorescence markers (Columns **2** & **4**). Producers in the CEN.PK2-1C (Row **A**) and TM6* (Row **D**) genetic backgrounds constitutively expressed mCherry (mCh) from the *GPD* promoter. *suc2*-deletion strains expressed either *eYFP* (in the CEN.PK2-1C background – Row **B**) or *eGFP* (in the TM6* background – Row **E**). These different markers enabled strains to be distinguished in mixed genotype populations (Rows **C** & **F**). Doublets were considered to be events where both genotypes were detected and so were added to both genotype frequency totals. Columns **1**-**2** show data as dot plots and **3**-**4** show the same data as contour plots to aid visualisation. Column **5** shows the frequencies of different regions in the associated row. The excitation laser was green (532 ± 5 nm) for *mCherry,* and was blue (488 ± 5 nm) for *eYFP* and *eGFP.* Emission was detected with orange (620/52 nm) for *mCherry*, yellow (583/26 nm) for *eYFP,* and green (525/30 nm) for *eGFP*.

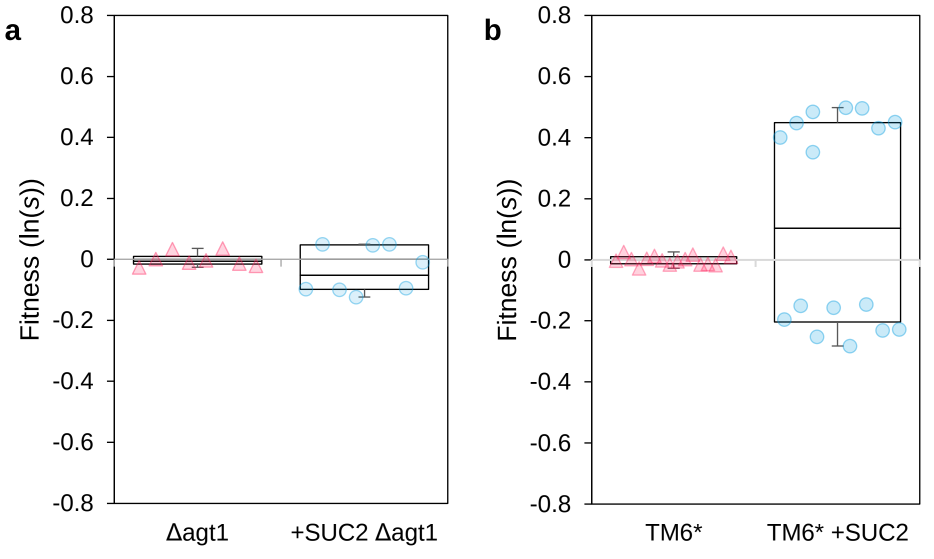

Supplementary Figure 15. To ensure that any fitness differences measured for the engineered strains in Figure 5 are the result of modifications to sucrose metabolism, equivalent competitions were conducted in 1% glucose media. Fitness differences in 1% sucrose media between wt, +SUC2 and +HXT7 (Fig. 3a) and between Δagt1 and +SUC2Δagt1 (Fig. 5) were lost in 1% glucose media (Supplementary Fig. 5a) and **a** (Welch's two-sided t-test: $t = 1.22$, $p = 0.258$, $n = 8$). **b** The competitions in 1% glucose with strains in the TM6* genetic background had significantly higher variation in fitness (Two sample F-test for variances (+SUC2Δagt1 c.f. TM6*+SUC2): $F = 19.6$, $p < 2.86 \times 10^{-4}$), despite not being significantly different overall (Welch's two-sided t-test (TM6* c.f. TM6*+SUC2): $t = 1.41$, $p = 0.178$). Although the source of this variation was not identified, when testing the fitness of strains in the TM6* genetic background, competitions in 1% glucose were run in parallel to the competitions in 1% sucrose so that this variation could be accounted for when calculating the fitness in Fig. 5 (see methods for more details). All data are provided in the Source Data File.

Source Data Files
Uncropped gel and blot scans:
Source: Supplementary Figure 1c (left)

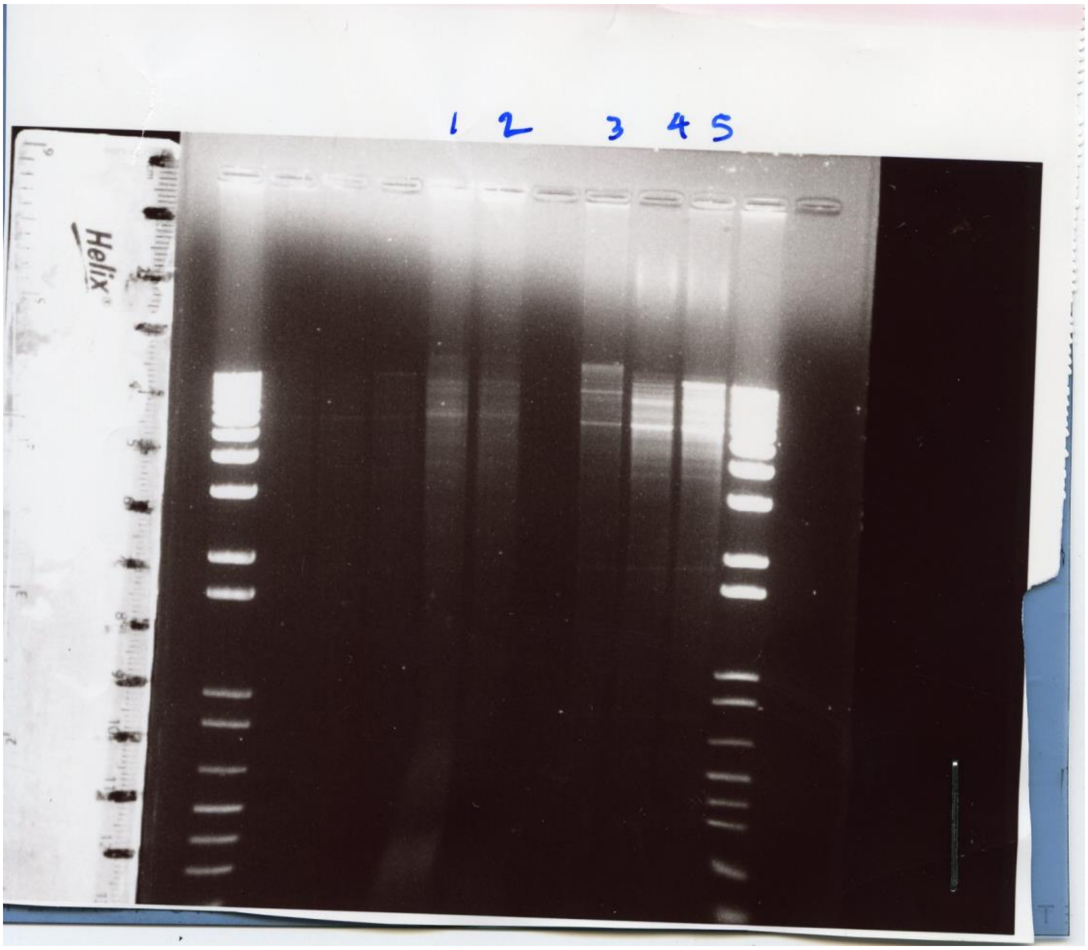

Uncropped gel and blot scans:

Source: Supplementary Figure 1c (right)

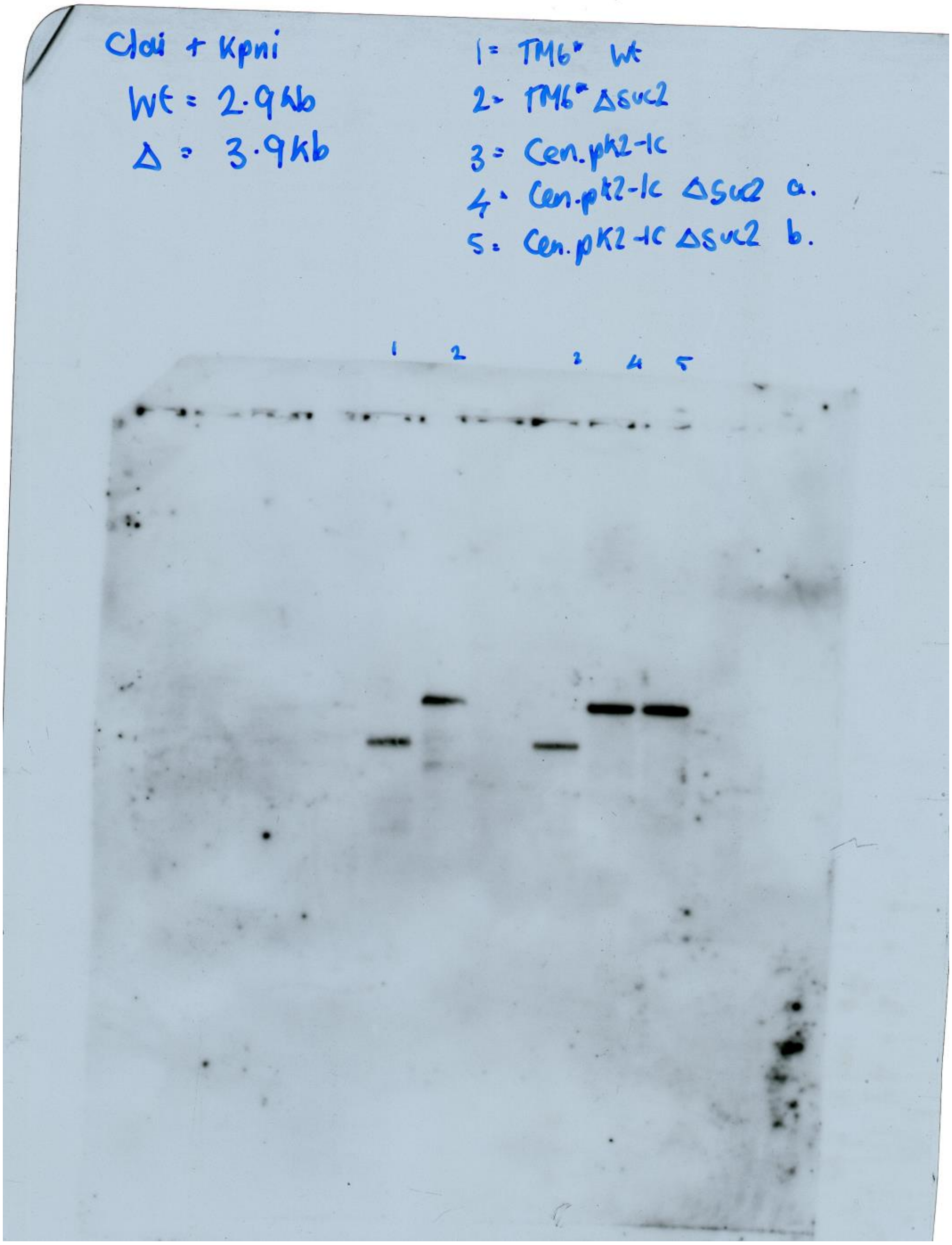

Uncropped gel and blot scans:

Source: Supplementary Figure 6

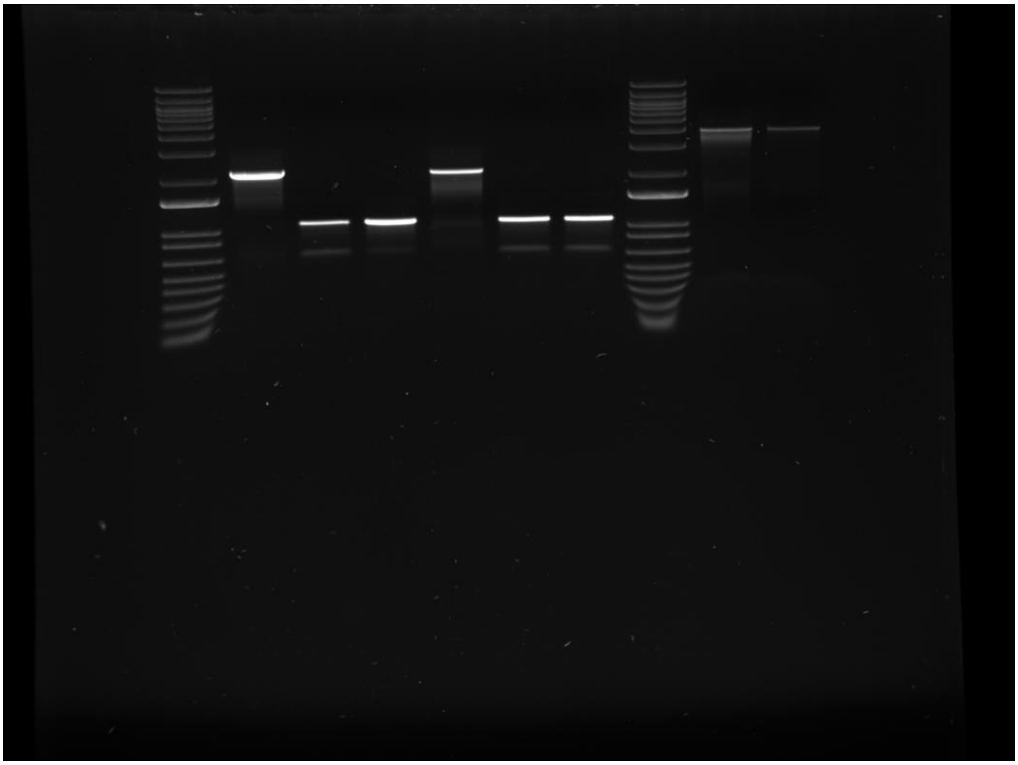

Uncropped gel and blot scans:

Source: Supplementary Figure 7 (left)

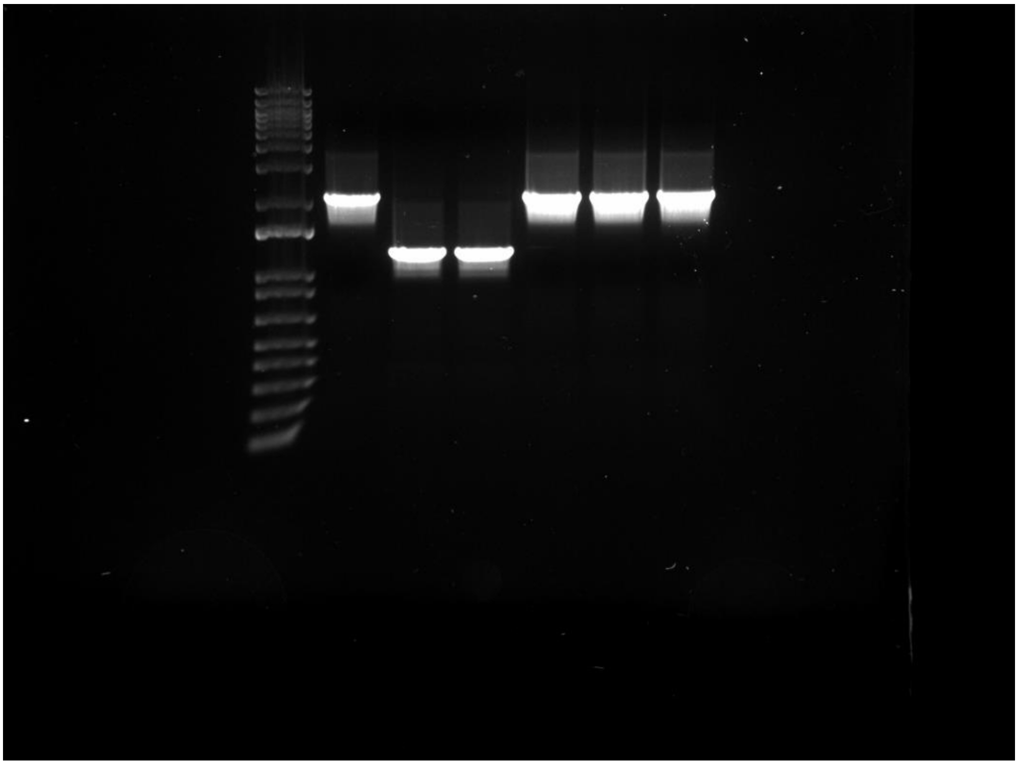

Source: Supplementary Figure 7 (right)

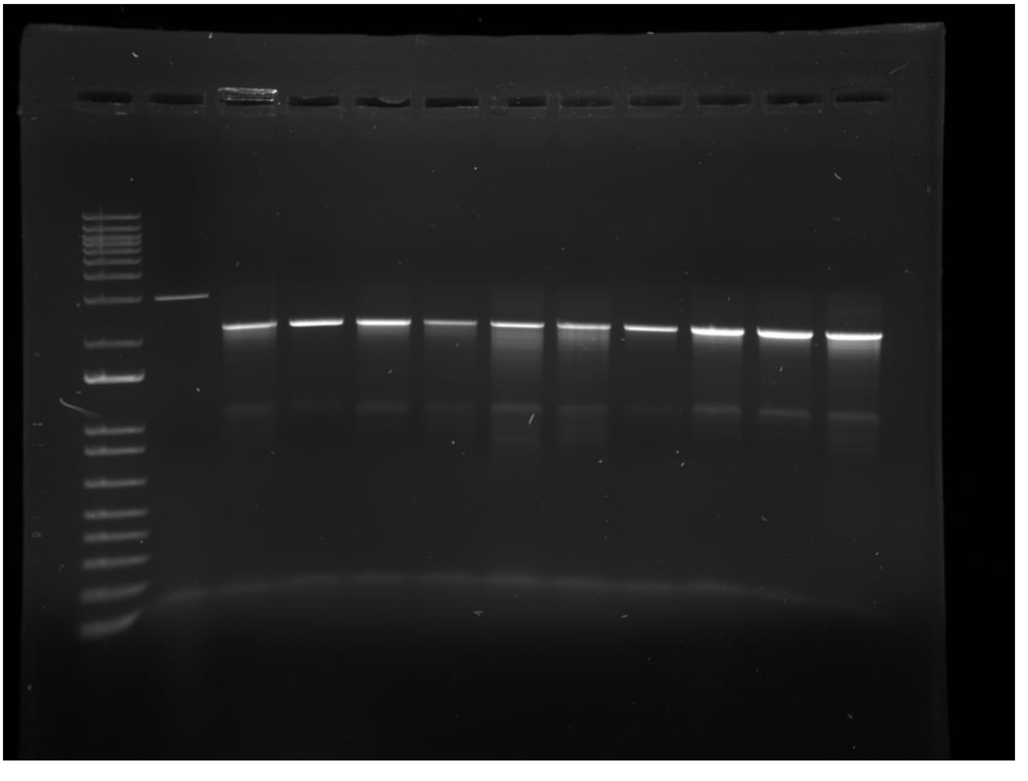

Supplement: Supplementary file 1 — Supplementary Information [file 41467_2024_52043_MOESM1_ESM.pdf]
