# Supplementary material for: Experimental evolution of yeast shows that public-goods upregulation can evolve despite challenges from exploitative non-producers
Source: Nat Commun. 2024 Sep 6;15:7810. doi: 10.1038/s41467-024-52043-9 (PMC11379824; doi:10.1038/s41467-024-52043-9)
Supplement: Supplementary file 3 — Description of Additional Supplementary Files [file 41467_2024_52043_MOESM3_ESM.pdf]

Description of Additional Supplementary Files

Supplementary Data 1: Data analysis for Figure 2. Normalised relative fitness values were compared against the ancestral WT using GLMs. Multiple comparisons of means of clones between resource environments were conducted using a clone: sugar interaction term and a post-hoc Tukey's (HSD method). Relative invertase activity was normalised against the ancestor and ln-transformed for statistical analysis using a GLM. To test the effect of initial evolution condition (from clonal population or in competition with non-producers), we performed a GLM with an interaction term between relative invertase activity and evolution condition (presence or absence of non-producers). See Supplementary Figure 3b for all replicates.

Supplementary Data 2: Data analysis for Figure 5 and Supplementary Fig. 12.

Supplementary Data 3: Details of strains used in this study.

Supplementary Data 4: Primer sequences used in this study. Underlined regions indicate complimentary regions for homologous recombination or In-fusion cloning.
