# Supplementary material for: Experimental evolution of yeast shows that public-goods upregulation can evolve despite challenges from exploitative non-producers
Source: Nat Commun. 2024 Sep 6;15:7810. doi: 10.1038/s41467-024-52043-9 (PMC11379824; doi:10.1038/s41467-024-52043-9)
Supplement: Supplementary file 5 — Reporting Summary [file 41467_2024_52043_MOESM5_ESM.pdf]

nature portfolio

Corresponding author(s): Ivana Gudelj

Last updated by author(s): Aug 7, 2024

Reporting Summary

Nature Portfolio wishes to improve the reproducibility of the work that we publish. This form provides structure for consistency and transparency in reporting. For further information on Nature Portfolio policies, see our Editorial Policies and the Editorial Policy Checklist.

Statistics

For all statistical analyses, confirm that the following items are present in the figure legend, table legend, main text, or Methods section.

n/a	Confirmed	
☐	☒	The exact sample size (n) for each experimental group/condition, given as a discrete number and unit of measurement
☐	☒	A statement on whether measurements were taken from distinct samples or whether the same sample was measured repeatedly
☐	☒	The statistical test(s) used AND whether they are one- or two-sided<br>*Only common tests should be described solely by name; describe more complex techniques in the Methods section.*
☐	☒	A description of all covariates tested
☐	☒	A description of any assumptions or corrections, such as tests of normality and adjustment for multiple comparisons
☐	☒	A full description of the statistical parameters including central tendency (e.g. means) or other basic estimates (e.g. regression coefficient) AND variation (e.g. standard deviation) or associated estimates of uncertainty (e.g. confidence intervals)
☐	☒	For null hypothesis testing, the test statistic (e.g. F, t, r) with confidence intervals, effect sizes, degrees of freedom and P value noted<br>*Give P values as exact values whenever suitable.*
☒	☐	For Bayesian analysis, information on the choice of priors and Markov chain Monte Carlo settings
☒	☐	For hierarchical and complex designs, identification of the appropriate level for tests and full reporting of outcomes
☒	☐	Estimates of effect sizes (e.g. Cohen's d, Pearson's r), indicating how they were calculated

Our web collection on statistics for biologists contains articles on many of the points above.

Software and code

Policy information about availability of computer code

Data collection	Spark 10M microplate reader (Tecan) used SparkControl V2.1 software (Tecan) and FLUOstar Omega (BMG) used Omega V3.00 software (BMG) to control growth conditions and make optical density readings from which population densities were calculated. Guava Easycyte 10HT was controlled using guavaSoft 3.2 and data was extracted using Guava InCyte software 3.2 (Merck Millipore).
Data analysis	R statistical software version 4.0.4 and MS Excel version 2308

For manuscripts utilizing custom algorithms or software that are central to the research but not yet described in published literature, software must be made available to editors and reviewers. We strongly encourage code deposition in a community repository (e.g. GitHub). See the Nature Portfolio guidelines for submitting code & software for further information.

Data

Policy information about availability of data

All manuscripts must include a data availability statement. This statement should provide the following information, where applicable:

- Accession codes, unique identifiers, or web links for publicly available datasets
- A description of any restrictions on data availability
- For clinical datasets or third party data, please ensure that the statement adheres to our policy

The data supporting this article can be found at https://doi.org/10.6084/m9.figshare.26206334. Source data are provided as a Source Data file.

Research involving human participants, their data, or biological material

Policy information about studies with human participants or human data. See also policy information about sex, gender (identity/presentation), and sexual orientation and race, ethnicity and racism.

Reporting on sex and gender	N/A
Reporting on race, ethnicity, or other socially relevant groupings	N/A
Population characteristics	N/A
Recruitment	N/A
Ethics oversight	N/A

Note that full information on the approval of the study protocol must also be provided in the manuscript.

Field-specific reporting

Please select the one below that is the best fit for your research. If you are not sure, read the appropriate sections before making your selection.

[x] Life sciences [ ] Behavioural & social sciences [ ] Ecological, evolutionary & environmental sciences

For a reference copy of the document with all sections, see nature.com/documents/nr-reporting-summary-flat.pdf

Life sciences study design

All studies must disclose on these points even when the disclosure is negative.

Sample size	For all experiments, n>=3, which is equivalent to similar published studies. These sample sizes were sufficient to detect statistically significant differences between test genotypes and/or conditions, where appropriate. Performing power analysis, a priori, was inappropriate for this study because detecting predetermined effect sizes were not sought.
Data exclusions	Data was not excluded from the analysis.
Replication	Experiments were performed in at least triplicate, with key experiments repeated 3-4 times to verify the reproducibility of empirical outcomes.
Randomization	Populations were randomly allocated to wells within microtitre plates, with starter cultures initiated from a randomly selected colony from an agar plate.
Blinding	Experimenters were not blinded during data collection or analysis because subjective measurements were not made.

Reporting for specific materials, systems and methods

We require information from authors about some types of materials, experimental systems and methods used in many studies. Here, indicate whether each material, system or method listed is relevant to your study. If you are not sure if a list item applies to your research, read the appropriate section before selecting a response.

Materials & experimental systems

n/a	Involved in the study	
[x]	[ ]	Antibodies
[x]	[ ]	Eukaryotic cell lines
[x]	[ ]	Palaeontology and archaeology
[ ]	[x]	Animals and other organisms
[x]	[ ]	Clinical data
[x]	[ ]	Dual use research of concern
[x]	[ ]	Plants

Methods

n/a	Involved in the study	
[x]	[ ]	ChIP-seq
[ ]	[x]	Flow cytometry
[x]	[ ]	MRI-based neuroimaging

Animals and other research organisms

Policy information about studies involving animals; ARRIVE guidelines recommended for reporting animal research, and Sex and Gender in Research

Laboratory animals	No laboratory animals were involved in this study
Wild animals	No wild animals were involved in this study
Reporting on sex	N/A
Field-collected samples	N/A
Ethics oversight	No ethical approval or guidance were required because the study involved harmless microorganisms.

Note that full information on the approval of the study protocol must also be provided in the manuscript.

Plants

Seed stocks	N/A
Novel plant genotypes	N/A
Authentication	N/A

Flow Cytometry

Plots

Confirm that:

- [x] The axis labels state the marker and fluorochrome used (e.g. CD4-FITC).
- [x] The axis scales are clearly visible. Include numbers along axes only for bottom left plot of group (a 'group' is an analysis of identical markers).
- [x] All plots are contour plots with outliers or pseudocolor plots.
- [x] A numerical value for number of cells or percentage (with statistics) is provided.

Methodology

Sample preparation	Laboratory strains of Saccharomyces cerevisiae cells were cultured in filter sterilized (0.2 micron) media and for flow cytometry the density of the culture was diluted appropriately in either the same media (omitting sugar) or in filter sterilized milli-Q water.
Instrument	Guava Easycyte 10HT (Merck Millipore)
Software	Guava InCyte software 3.2 (Merck Millipore)
Cell population abundance	5000 events were acquired per replicate for all competition experiments. 4388-5000 events were acquired for each genotype/condition combination for estimating HXT2 expression. For measuring growth rates at very low population densities, 5000 events were acquired or the number of events in 123.9 microlitres were acquired for those that had not reached 5000 events within that volume. Reagents used for culturing cells for experiments that used flow cytometry were filter sterilised with a 0.2 micron syringe filter to remove particulates that may compromise the purity of measurements. Strains were distinguished based on their fluorescent protein properties as detailed in Supplementary Figure 14.
Gating strategy	Yeast populations were gated on FSC and SSC to specify cells from other detected particles. This gated more than 95% of detected events which were assessed for fluorescence. Excitation lasers were green (532 ± 5 nm) for mCherry, and blue (488 ± 5) for eYFP/E. Emission was detected with green (525/30 nm) for EGFP, orange (620/52 nm) for mCherry, and yellow (583/26 nm) for eYFP. To distinguish strains in mixed populations, for every time point an axenic population of each genotype was measured as a reference. Example plots and data of this strategy is detailed in Supplementary Figure 14.

- [x] Tick this box to confirm that a figure exemplifying the gating strategy is provided in the Supplementary Information.
